# Implementation of the fifth link of the Chain of Survival concept for out-of-hospital cardiac arrest

**DOI:** 10.1186/cc10873

**Published:** 2012-03-20

**Authors:** T Tagami, R Tosa, M Omura, H Yokota, H Hirama

**Affiliations:** 1 Nippon Medical School, Tokyo, Japan; 2 Aizu Chuo Hospital, Fukushima, Japan

## Introduction

The 2010 resuscitation guidelines of the American Heart Association-International Liaison Committee on Resuscitation recommend an additional fifth link (post-resuscitation care in a regional center) in the Chain of Survival concept for out-of-hospital cardiac arrest (OHCA) in addition to early access to emergency medical care (first link), early cardiopulmonary resuscitation (second link), early defibrillation (third link), and early advanced cardiac life support (fourth link). However, no direct evidence supports its implementation. Our study aimed to determine the effectiveness of this fifth link.

## Methods

This multicenter, region-wide, prospective clinical study involved all eligible OHCA patients in the Aizu region (*n *= 1,482, suburban/rural, Fukushima, Japan). Primary outcomes before (January 2006 to April 2008) and after (January 2009 to December 2010) the implementation of the fifth link were evaluated. After implementation, all post-cardiac arrest syndrome patients were concentrated in a hospital having facilities for post-resuscitation management and provided intensive care, including appropriate hemodynamic and pulmonary management, therapeutic hypothermia, and percutaneous coronary intervention. The primary outcome measure was patient survival at 1 month with a favorable neurological outcome.

## Results

The primary outcome improved significantly from 0.5% (before, 4/770) to 3.0% (after, 21/712) (*P *< 0.0001). The multivariate odds ratio for the primary outcome was 8.3 (95% CI, 2.6 to 26.6) after the implementation of the fifth link, 7.1 (CI, 2.0 to 25.1) for a bystander-witnessed arrest, and 5.0 (CI, 2.6 to 26.6) for early defibrillation.

## Conclusion

The proportion of OHCA patients with a favorable neurological outcome improved significantly after the implementation of the fifth link of the Chain of Survival. This finding may require confirmation in an urban setting and/or with randomized trials.

## Trial registration

University Hospital Medical Information Network Clinical Trials Registry: UMIN000001607 [http://apps.who.int/trialsearch/trial.aspx?trialid=JPRN-UMIN000001607]

